# Modeling-Based Assessment of 3D Printing-Enabled Meniscus Transplantation

**DOI:** 10.3390/healthcare7020069

**Published:** 2019-05-10

**Authors:** Zimeng Zhang, Qian Wu, Li Zeng, Shiren Wang

**Affiliations:** Department of Industrial and Systems Engineering, Texas A&M University, College Station, TX 77843, USA; zimeng_zhang@tamu.edu (Z.Z.); hi_qianwu@tamu.edu (Q.W.); lizeng@tamu.edu (L.Z.)

**Keywords:** 3D printing, Markov model, meniscus transplantation, pathway model

## Abstract

3D printing technology is able to produce personalized artificial substitutes for patients with damaged menisci. However, there is a lack of thorough understanding of 3D printing-enabled (3DP-enabled) meniscus transplantation and its long-term advantages over traditional transplantation. To help health care stakeholders and patients assess the value of 3DP-enabled meniscus transplantation, this study compares the long-term cost and risk of this new paradigm with traditional transplantation by simulation. Pathway models are developed to simulate patients’ treatment process during a 20-year period, and a Markov process is used to model the state transitions of patients after transplantation. A sensitivity analysis is also conducted to show the effect of quality of 3D-printed meniscus on model outputs. The simulation results suggest that the performance of 3DP-enabled meniscus transplantation depends on quality of 3D-printed meniscus. The conclusion of this study is that 3DP-enabled meniscus transplantation has many advantages over traditional meniscus transplantation, including a minimal waiting time, perfect size and shape match, and potentially lower cost and risk in the long term.

## 1. Introduction

The menisci play a critical role in the knee in terms of load transmission, weight bearing, shock absorption, lubrication and joint stabilization [1,2,3,4,5], and meniscus treatments are imperative for the knee [3,6]. Meniscus allograft transplantation is one of the treatments for symptomatic patients with a meniscus-deficient knee [7]. It can relieve pain and improve knee function in the short term and delay the deterioration of the knee to total knee arthroplasty (TKA) in the long term. However, problems exist in traditional donor-based meniscus transplantation. A major problem is the shortage of available meniscal tissue supply for such transplantations due to limited musculoskeletal tissue donation [8]. The donor shortage leads to an extended waiting time for a suitable allograft, which may deteriorate the patient’s condition and increase the chance of entering TKA. Statistics show that about a quarter of patients died while waiting for a donor [9]. In addition, there exists the risk of disease transmission with allografts and failures caused by donor–recipient shape mismatch [10,11].

The advent of tissue engineering provides an alternative way to treat torn meniscus [7]. It involves using functional biomaterials as scaffolds and inducing cell differentiation on the scaffolds to engineer tissue substitutes. Briefly, biomaterials are synthesized and used to fabricate scaffolds which are porous matrices. Fully functionalized tissues are formed by seeding relevant cells onto the scaffolds and adding growth factors for cell proliferation and differentiation. Traditional methods to fabricate scaffolds, such as chemical/gas foaming [12], solvent casting [13,14], freeze drying [15], and phase separation [16], suffer from limited ability to construct complex structures with specific dimensions.

Three-dimensional (3D) printing is a promising way to fabricate artificial menisci with controllable size and structures [17]. In general, 3D printing makes objects in a layer-by-layer fashion based on 3D model data and thus enables the production of customized medical products for individual patients. At the beginning of this century, 3D printing was applied in the medical field for fabricating dental implants and custom prosthetics [7]. In 2003, the first bioprinter was developed and biological materials, such as cells, biomaterials and growth factors, were used as bioinks for tissue/organ fabrication [18]. The terms “bioprinting” and “biofabrication” were created. Since then, many studies have demonstrated the proof-of-concept of tissue engineering [7,19]. In 2015, meniscus scaffolds with desired mechanical properties were successfully fabricated using 3D printing, which lays a foundation for the development of meniscus substitutes [20]. Moreover, after many years of endeavors in 3D printing, biomedical engineering and materials science, clinical applications of 3D-printed implants were achieved [21,22]. For example, a baby with tracheobronchomalacia was cured using a 3D-printed bioresorbable tracheal splint [21]. Patient-specific biodegradable scaffolds using polycaprolactone (PCL) were printed and implanted for maxillofacial bone reconstruction [22]. 3D-printed artificial skin was implanted in an animal study and will soon undergo clinical trials [23].

**Figure 1:**
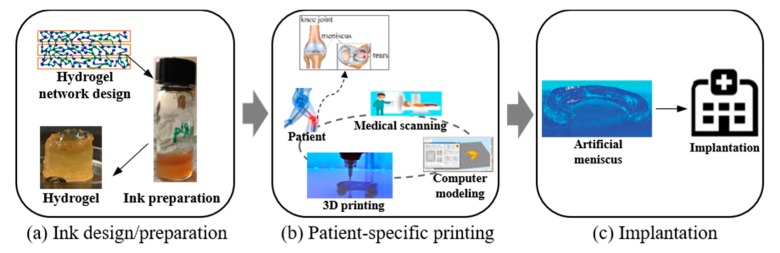
Roadmap for 3D printing of an artificial meniscus and meniscus implantation.

The roadmap for 3D printing of an artificial meniscus is illustrated in Figure 1. First, biomaterials with specific functionalities are designed and prepared, as shown in Figure 1a. The specific dimensions of the patient’s meniscus can be obtained through medical scanning and the computer model of the meniscus will be generated based on the results. Then, the prepared biomaterials are loaded into the cartridge and 3D printing starts. The printed biomaterials will be cross-linked and hydrogels (with a network of insoluble polymers that expand in water) will be formed to secure the dimensions as printed. The printing process is shown in Figure 1b. The printed meniscus will then be implanted in the patient’s body, as shown in Figure 1c.

The incorporation of 3D printing in tissue engineering will lead to a new paradigm for meniscus transplantation, where 3D-printed meniscus substitutes are used in place of donated allografts. This 3D printing-enabled (called 3DP-enabled hereafter) meniscus transplantation can solve the problems of traditional transplantation. Specifically, 3D-printed meniscus will reduce patients’ waiting time for transplantation and thus prevent their conditions from deteriorating. The customized meniscus made by 3D printing matches well with the patient’s original meniscus, which can avoid the pain caused by friction due to meniscus mismatch. There are also potential benefits in terms of cost and risk for patients: 3D-printed meniscus is likely to be cheaper than the meniscus allograft, thus reducing the transplantation cost, and the risk of developing TKA could be reduced because of the timely treatment provided by 3DP-enabled meniscus transplantation [24,25].

To help health care stakeholders and patients assess the value of 3DP-enabled meniscus transplantation, we compare 3DP-enabled and traditional meniscus transplantations in terms of long-term cost and risk for patients by simulation in this study. Pathway models of the two care processes are established, which include a Markov process to model the patient’s state transition after transplantation. Through simulation, cost and risk for patients are estimated and compared. The contribution of this work lies in providing a modeling-based approach for assessing 3D printing-enabled transplantation, which integrates pathway modeling, Markov modeling, and available data on traditional transplantation and 3D printing of implants. Moreover, quality of 3D-printed meniscus is considered in the modeling, in order to give a comprehensive picture of this new paradigm and generate useful insights for researchers in this field.

## 2. Methods

### 2.1. Model

**Figure 2:**
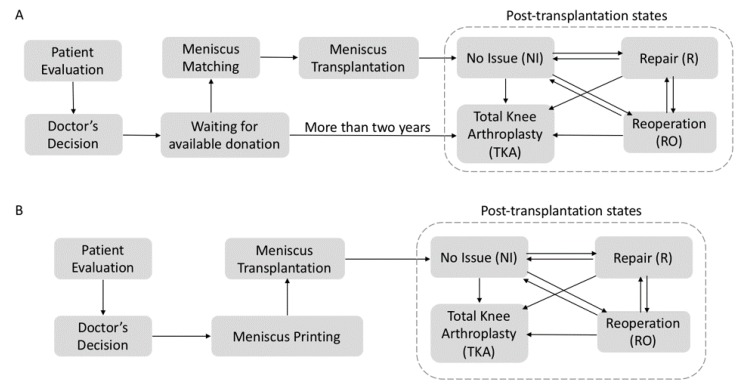
Pathway models of meniscus transplantation: (**A**) traditional vs. (**B**) 3DP-enabled.

In order to estimate the long-term cost and risk for patients undergoing traditional versus 3DP-enabled meniscus transplantation, we create pathway models of the two types of transplantation, as shown in Figure 2, and simulate a 20-year period of the system to monitor patients in the pathway models. The simulation period is long enough for this study considering that the reported lifespan of a meniscus allograft transplantation ranges from as short as 2 months to as long as 17.3 years [26,27,28]. The short lifespan of meniscus allografts is due to significant varus malalignment after transplantation which requires valgus treatment [26]. In the following, we first describe the pathway model of traditional transplantation and then explain how the 3DP-enabled transplantation model differs.

In the traditional paradigm as shown in Figure 2A, the patient is first evaluated by the doctor to decide whether to have a meniscus transplantation. If the patient needs a transplantation, he/she will enter into the queue waiting for a matching meniscus allograft.

The state transition of the patient after transplantation is modeled by a Markov process. Specifically, we define the possible treatments that the patient receives as four states, namely, no issue (NI), repair (R), reoperation (RO), and TKA. NI represents the state where no special treatment needs to be performed. R represents the state where minor treatment needs to be performed on the meniscus. RO represents the state where postoperative conditions like arthrofibrosis occur, and subsequently, a new operation needs to be performed. TKA is the most severe state a patient can experience, where a surgical procedure is performed to replace part of the knee joint. Among the four states, R and RO are defined as “intermediate” states.

The initial state of the patient immediately after meniscus allograft surgery is defined as NI. After that, each year is counted as a period, and the state transition takes place after the annual check. In each state, the patient has certain probabilities to transfer to other states.

The 3DP-enabled pathway model shown in Figure 2B is similar to the traditional model in most steps except for a major difference: a patient who needs a transplantation will not be placed in a queue to wait for meniscus donors. Instead, a 3D-printed meniscus that matches with the patient will be prepared immediately for his/her transplantation.

### 2.2. Assumptions

In the above pathway models of meniscus transplantation, the assumptions made to run the simulation and their justifications are given below:

1. The arrival of patients to the waiting queue follows an exponential distribution with an average interarrival time of 1 week; the arrival of donors follows a mixed exponential distribution with the average interarrival time being a random sample from uniform (1,5). Here, a random interarrival time is used to reflect the uncertainty in donor arrivals that exists in reality [29].
2. The queue waiting for allografts in the traditional transplantation model follows the first-come, first-served rule [30]. The capacity of the queue is set to infinity. If a patient stays in the queue for too long (exceeding the threshold of waiting time), it is reasonable to assume that the condition of the meniscus will deteriorate, forcing the patient to have TKA.
3. A delay in transplantation caused by other factors, such as acquiring the donated meniscus and transportation in traditional transplantation, or access to a printer and preparation of the 3D-printed meniscus in 3DP-enabled transplantation, are negligible compared to the waiting time for an allograft (months or years) [31], so those factors are ignored in this study.
4. The patients have no pre-existing osteoarthritic changes, synovial disease, or inflammatory arthritis. This population comprises the most typical candidates for meniscus allograft and has been considered in related studies (e.g., [32]).
5. Due to the low complication rates reported in the literature [33,34], the transplantation procedure is assumed to be successful, without postoperative deep vein thrombosis, infection, or allograft rejection [35].
6. After the initial transplantation, a patient can enter the intermediate states (R or RO) twice at most; if he/she enters that kind of state for the third time, then he/she will be forced to TKA. This assumption is similar to what is used in existing studies [4,36], which leads to a survival rate of meniscus transplantation in our model close to the ones reported in the literature.

### 2.3. Model Parameters of Traditional Meniscus Transplantation

In the traditional transplantation model, once the patient obtains a suitable allograft, he/she will have the initial transplantation. The threshold of waiting time to enter TKA is 2 years or 104 weeks. That is, if a patient stays in the queue for 2 years, he/she will automatically enter TKA. This threshold is chosen because studies show that meniscal treatment failures start to occur after 2 years, with the mean of occurrences ranging from 2.2 to 4.2 years postoperatively [37]. After the initial transplantation, the patient undergoes an annual check that determines his/her next state (NI, RO, R or TKA).

The total cost for a patient in traditional meniscus transplantation is calculated by:

Cost per patient = patient evaluation cost + waiting cost + transplantation cost + post-transplantation cost(1)

In the above cost model, the evaluation cost is ignored in our calculation as it is usually very small. The waiting cost is proportional to the patient’s waiting time in the queue and generated weekly for each patient. The meniscus transplantation cost (US $8875) includes the meniscus allograft cost, surgery cost, and physical therapy and procedure costs [4,36]. The post-transplantation cost refers to subsequent treatment costs for RO, R and TKA after the initial meniscus transplantation, which covers costs of the operation procedure, related physical therapies and meniscus allograft in the case of TKA. The cost parameters in the simulation for traditional meniscus transplantation are listed in Table 1 with their sources cited.

**Figure 3:**
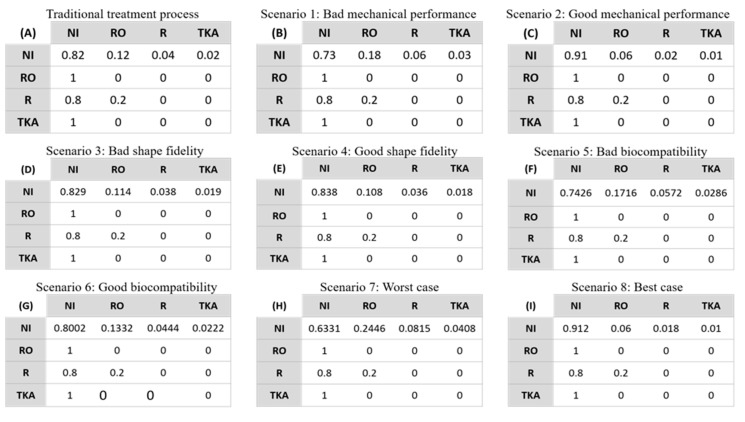
Post-treatment transition matrix: traditional transplantation (**A**) and eight scenarios in 3DP-enabled transplantation (**B–I**).

Another set of parameters is the probability transition matrix of the Markov model. Table 2 displays the estimated probabilities of each state and transitions between the states according to related literature, as given in the table. Based on those estimates, an appropriate value is selected for each probability. The corresponding transition matrix is given in Figure 3A.

### 2.4. Model Parameters of 3D Printing-Enabled Transplantation

Since 3D printing-enabled meniscus transplantation has not been available in clinical practice, the parameters of its pathway model are determined based on relevant literature. All data used in the following calculations are from studies that were approved by the institutional review board (IRB) and satisfy other related regulatory requirements.

The cost model of 3DP-enabled transplantation is:

Cost per patient = patient evaluation cost + transplantation cost + post-transplantation cost(2)

Note that there is no waiting cost in the above model. Again, we ignore the patient evaluation cost as it is small. The transplantation cost includes the 3D-printed meniscus cost, i.e., all costs for preparing the 3D-printed meniscus, surgery cost, and associated therapy cost. The post-transplantation cost is calculated in the same way as in the traditional model, with the transition matrix having different probabilities.

In contrast to traditional meniscal allografts, there is a lack of cost estimates on 3D-printed meniscus implants due to the current exploratory stage of tissue engineering and 3D bioprinting technology. In previous reports, the cost of 3D printing-based soft tissue prototype prosthesis was analyzed by adding the material cost, facility cost and labor cost together [42]. Similarly, in this study, we estimate the cost of the 3D-printed meniscus according to the structure of the human meniscus and the materials needed for manufacturing a meniscus implant. Costs of cells, cell culture and growth factors, which are important in preparing the implant, are also considered. Based on the literature [36] and advice from researchers in the biomedical field, we obtain an estimate of the 3D-printed meniscus cost, which is about $3124 per implant. A detailed description of the types of materials/amount of each ingredient and corresponding costs is listed in Table 3. Consequently, the transplantation cost is $7249, which is obtained by replacing the meniscus allograft cost ($4750) in traditional transplantation cost ($8875) with the 3D-printed meniscus cost.

Since research on 3D-printed tissues is still in the explorative or clinical testing stage, there are no follow-up studies available for possible conditions of patients after 3D printing-based meniscus transplantation. Therefore, we estimate the probability transition matrix by modifying the probabilities in the traditional model. Our modification is based on the belief that the difference of state/transition probabilities between traditional transplantation and 3DP-enabled transplantation mainly depends on quality of 3D-printed meniscus compared to meniscus allograft; quality of 3D-printed meniscus can be evaluated in terms of three aspects, namely, mechanical performance, shape fidelity, and biocompatibility.

In the original case, 3D-printed meniscus is similar to allograft in those three aspects, and thus the same transition matrix as in the traditional model will be used. However, in practice, 3D-printed meniscus is likely to differ from allograft to some extent. To quantify the uncertainty in the quality of 3D-printed meniscus implants, we adjust the probabilities in the original transition matrix based on the three above-mentioned factors. Specifically, we consider two scenarios (good and bad) of each factor to reflect possible typical cases of quality of 3D-printed meniscus. Since no direct quantitative analysis of the factors exists in the literature, we make use of available data and make some reasonable assumptions to estimate their effects on the cost and risk in 3DP-enabled meniscus transplantation. Moreover, we consider two scenarios to show their simultaneous effect. Details of the eight scenarios are described and the corresponding transition matrices are listed in Figure 3.

For mechanical performance, the natural meniscus shows a compression strength in the range of 5–40 MPa [48]. For the 3D-printed meniscus, the reported compression strength exhibits a wide range from several MPa to 93.5 MPa [20,41,49,50,51,52]. With higher mechanical performance, the probabilities of R, RO and TKA decrease and the probability of NI increases compared to the original case and vice versa. So, two cases are considered for this factor: the R, RO and TKA probabilities are 150% (Scenario 1, representing bad mechanical performance) or 50% (Scenario 2, representing good mechanical performance) of those in traditional transplantation. The corresponding transition matrices are shown in Figure 3B,C.

For shape fidelity, the R, RO and TKA probabilities of the bad and good cases are calculated in a similar way to those of mechanical performance. The meniscus allograft has a 5–10% chance of mismatch with the patient, while the 3D-printed meniscus can achieve the exact same shape as the patient’s meniscus [53]. Current technology can reach a resolution as high as 100 μm using an inkjet dispenser printing technique and nanoscale printing via two-photon-polymerization (2PP) [54]. With a more precise shape of the 3D-printed meniscus, the probabilities of R, RO and TKA decrease compared to the original probabilities in traditional transplantation. So, we adjust those probabilities to 95% (Scenario 3, representing bad shape fidelity) or 90% (Scenario 4, representing good shape fidelity). The corresponding transition matrices are shown in Figure 3D,E.

For biocompatibility, higher chances of immunology rejection are expected for engineered meniscus implants compared to the case of allografts. We define cell viability as the indicator of biocompatibility of the engineered tissue substitutes. Reported cell viability is in a range of 62.7–97% [11,55]. So, we adjust the probabilities of R, RO and TKA to 143% (Scenario 5, representing bad biocompatibility) or 111% (Scenario 6, representing good biocompatibility). The corresponding transition matrices are shown in Figure 3F,G.

For the simultaneous effect of these three factors, we integrate each factor’s influence on the quality of a 3D-printed meniscus to form two extreme cases (best and worst). Specifically, by multiplying all the probability values in the bad scenarios (i.e., probabilities adjusted to 150%, 95% and 143% for mechanical performance, shape fidelity and biocompatibility, respectively), the worst case (Scenario 7) is obtained where the probabilities of R, RO and TKA are tuned to 203.78%. Similarly, by multiplying all the probability values in the good scenarios (i.e., probabilities adjusted to 50%, 90% and 111% for mechanical performance, shape fidelity and biocompatibility, respectively), the best case (Scenario 8) is obtained where the probabilities of R, RO and TKA are tuned to 49.95%. The corresponding transition matrices are shown in Figure 3H,I.

### 2.5. Simulation Outputs

To estimate the long-term cost and risk for patients, we simulate the pathways of 1040 patients over a 20-year period for each type of meniscus transplantation. The averages of 10 runs are used as final results. Specifically, patients are generated one by one following the assumed distributions, and the state and associated cost of each patient are monitored. For each year during the 20-year period, the cumulative number of patients in TKA, i.e., the total number of patients in TKA over all the past years, and the cumulative gross cost for patients, i.e., the total cost for patients over all the past years, are recorded.

There are three outputs from the simulation: (1) the cumulative number of patients in TKA due to allograft waiting, (2) the cumulative gross cost for patients, and (3) the cumulative total number of patients in TKA. The number of patients in TKA measures the risk of meniscus transplantation, and the cumulative gross cost measures the economic load for the health care system. These measures will help health care stakeholders and patients to assess the value of 3DP-enabled transplantation compared to traditional transplantation. Moreover, they will reveal potential benefits of 3D-printed meniscus and suggest future directions of 3D bioprinting and tissue regeneration research.

We also conduct a sensitivity analysis on 3DP-enabled meniscus transplantation to investigate how the uncertainty in the quality of 3D-printed meniscus affects the long-term cost and risk in this paradigm. Specifically, the cumulative gross cost for patients and the cumulative total number of patients in TKA will be calculated under the above-mentioned eight scenarios which represent bad and good qualities of the printed meniscus.

## 3. Results

### 3.1. Number of Patients in TKA

**Figure 4:**
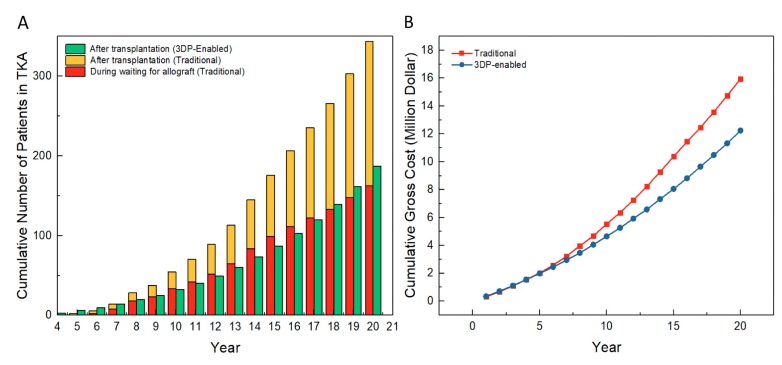
Comparison of traditional and 3DP-enabled meniscus transplantation: (**A**) Cumulative number of patients in TKA and (**B**) Cumulative gross cost for patients.

Figure 4A reports the number of patients in the TKA state in traditional and 3DP-enabled meniscus transplantation. In traditional transplantation, TKA occurs either during the period of waiting for a matching allograft (up to 2 years) or during the period after initial transplantation (20 years). The first case does not apply for 3DP-enabled transplantation. We can see that the number of patients in TKA due to allograft waiting accounts for nearly 50% of the total number of patients in TKA in traditional transplantation. The number of patients in TKA in 3DP-enabled transplantation is around one-third of the total number of patients in TKA in traditional transplantation. This indicates that patients have a much lower chance of developing TKA in 3DP-enabled transplantation. It is worth mentioning that a smaller number of TKAs implies not only a lower risk of severe conditions, but also a huge saving in cost.

### 3.2. Cost Analysis

Figure 4B reports the annual cumulative gross cost for all patients during the simulated period. Note that the same transition matrix is used in the traditional and 3DP-enabled models. The result shows a clear economic benefit for 3DP-enabled transplantation. For example, the cumulative gross cost of 3DP-enabled transplantation (~12.4 million dollars) is considerably smaller than that of traditional transplantation (~15.9 million dollars) in the final year. Two major factors that we believe contribute to the savings are that the 3D-printed meniscus implant is less expensive, and that without waiting for an allograft, a patient has no chance of entering TKA before transplantation.

### 3.3. Sensitivity Analysis

**Figure 5:**
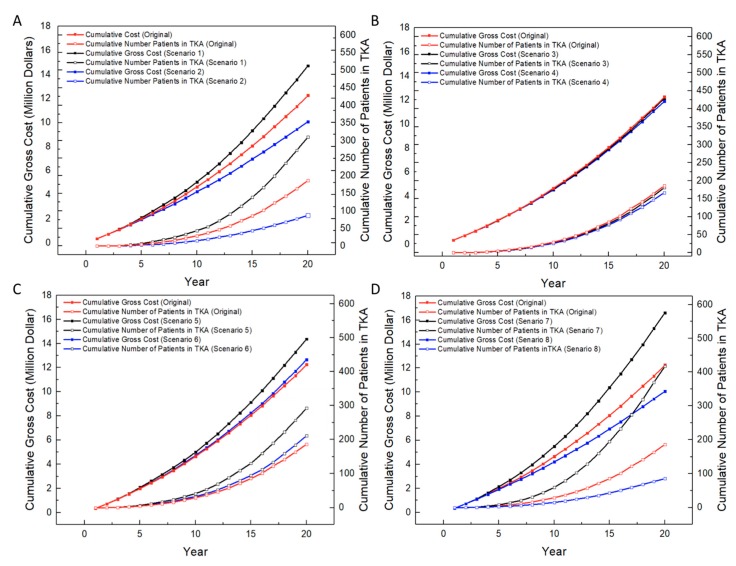
Sensitivity analysis on 3DP-enabled meniscus transplantation: (**A**) Effect of mechanical performance, (**B**) Effect of shape fidelity, (**C**) Effect of biocompatibility, and (**D**) Simultaneous effect of mechanical performance, shape fidelity and biocompatibility.

Figure 5 shows the results of the sensitivity analysis on 3DP-enabled transplantation under the eight scenarios given in Figure 3. In each plot, “original” represents the case where the transition matrix is the same as that of traditional transplantation; in other words, the 3D-printed meniscus has a similar quality to the allograft. The number of patients in TKA and the cumulative gross cost for patients are calculated in the “original” case and for the cases representing good or bad quality of a 3D-printed meniscus.

#### 3.3.1. Effect of Mechanical Performance of 3D-Printed Meniscus

According to Figure 5A, the mechanical performance of the 3D-printed meniscus has a fairly significant effect on the cumulative gross cost and the number of patients in TKA. Compared to the original case, in the final year, the cost increases by about 2 million when the mechanical performance is bad, and decreases by about 2 million when the mechanical performance is good; the number of patients in TKA increases by about 120 in the bad case and decreases by about 100 in the good case. It is interesting to notice that the increase in the number of patients in TKA in the bad case speeds up around Year 10. This is probably because the bad quality of the 3D-printed meniscus implant has forced many patients into the TKA state.

#### 3.3.2. Effect of Shape Fidelity of 3D-Printed Meniscus

Figure 5B shows that shape fidelity has a small effect on the cumulative gross cost and the number of patients in TKA. The cost is similar to that in the original case when the shape fidelity of the 3D-printed meniscus implant is good (~11.9 million dollars) and when it is bad (~12.1 million dollars); the number of patients in TKA in the good case (~166 patients) is about 20 fewer than that in the original case.

#### 3.3.3. Effect of Biocompatibility of 3D-Printed Meniscus

Figure 5C shows that the cumulative gross cost and the cumulative number of patients in TKA when the biocompatibility of 3D-printed meniscus implants is good are close to those in the original case; in the case of bad biocompatibility, however, there is a ~26.5% increase in the cumulative gross cost and a significant increase (~111%) in the cumulative number of patients in TKA (~292 patients) in the final year. This suggests that a low-quality 3D-printed meniscus would result in a higher chance for the patient to enter TKA and hence a higher cost.

#### 3.3.4. Simultaneous Effect of Mechanical Performance, Shape Fidelity and Biocompatibility

Figure 5D illustrates the simultaneous effect of the three factors. The trend of the cumulative gross cost for patients and the cumulative number of patients in TKA is consistent with the individual studies in Scenarios 1–6, except that the differences are larger due to the combined effect of the factors. Specifically, when 3D-printed meniscus implants are in best quality, the cumulative gross cost decreases by ~4.34 million dollars and the number of patients in TKA reduces by ~207 in the final year. In the worst case, the cost increases by ~2.2 million dollars and the number of patients in TKA increases by ~125 in the final year. This suggests that high mechanical strength, precise shape dimension and good biocompatibility of 3D-printed meniscus lead to a lower cost and a lower chance for patients to enter TKA.

It is worth mentioning that both the cumulative gross cost and the cumulative number of patients in TKA are exponentially increasing every year in all scenarios, and the rate of increase also increases over time. The exponential trend can be explained by the fact that the chance of entering TKA and thus cost increase over time. A general conclusion of the sensitivity analysis is that these three factors have a significant effect on the cost and risk of 3DP-enabled meniscus transplantation; the benefits of this new paradigm rest on good quality of 3D-printed meniscus implants; with low-quality meniscus implants, 3DP-enabled meniscus transplantation may have no advantage over traditional transplantation. From this point, a high-quality meniscus with similar properties to a natural meniscus is highly needed for substantial benefits to be gained from the 3D printing technology.

## 4. Discussion

In this paper, pathway models of 3DP-enabled and traditional meniscus transplantations are established, and a simulation based on the models is conducted to estimate their cost and risk over a 20-year period. Our simulation results indicate that adapting 3D printing technology to meniscus transplantation can potentially bring down the cost and risk of TKA for patients in the long term.

Note that, as mentioned previously, there is no study about 3D-printed meniscus transplanted in the human body to the best of our knowledge. 3D printing of soft tissues for humans is still in an exploratory stage, and it is premature to conclude that 3D-printed meniscus has an advantage of cost over traditional allograft transplantation. Nonetheless, the purpose of this paper is as an early stage reference for researchers in the related areas to arouse awareness about the similarities and differences between traditional and 3D-enabled transplantation. Moreover, patient acceptability, which is defined as the ability and willingness of patients to use a medical procedure, is critical to the incorporation of 3D printing technologies in health care [56]. Quantifying the potential benefits of 3DP-enabled meniscus transplantation in terms of cost and risk and revealing its other unique advantages, such as the elimination of waiting time and matching for a suitable allograft and the perfectly customized meniscus structure which can avoid many postoperative problems, will help promote patient acceptability of this new, promising paradigm.

3D printing is believed to have broader influence on the health field. There are many other tissues/organs of the human body, e.g., skin, blood vessel, liver, kidney, heart, etc., for which allografts are much more difficult to obtain and a long waiting time is more deadly to patients. The maturity of the 3D printing technique is expected to play a revolutionary role in changing the whole picture of these areas.

Another aspect that cannot be overlooked is the cost of tissue/organ allografts. As the demand for allografts increases, so too does their associated cost. The materials for 3D printing, on the other hand, are easily available and expected to become less expensive in the future with the advancement of 3D printing technology. Further, a more economical means of tissue engineering will be achieved when a 3D printing-based cyber manufacturing system is established in which a customized artificial tissue/organ is fabricated in a 3D printing center and delivered to the hospital of the patient.

The biggest challenge in our modeling process is the lack of data from follow-up studies about 3D-printed meniscus. Therefore, for the purpose of this research, reasonable assumptions have been made as needed based on literature. The model setup and simulation results will be updated when real data and more related information become available.

For possible future directions, the cost structure of 3D-printed meniscus will be further studied to improve the precision and comprehensiveness of the cost analysis. Efforts will also be made to investigate the cost and risk of 3DP-enabled transplantation for other tissues/organs which require more advanced development of 3D printing technology.

## Figures and Tables

**Table 1:** List of parameters in the simulation of traditional meniscus transplantation.

Parameter	Value	Unit	Source
Cost of no issue (NI)	0	$ per person	Zhang et al. [36]
Cost of repair (R)	2760	$ per person	Ramme et al. [4]
Cost of reoperation (RO)	1770	$ per person	Ramme et al. [4]
Cost of total knee arthroplasty (TKA)	14,167	$ per person	Ramme et al. [4]
Cost of waiting	30	$ per person per week	Assumption
Cost of meniscus transplantation	8875	$ per person	Ramme et al. [4]

**Table 2:** List of state/transition probabilities in traditional meniscus transplantation.

State/Transition	Estimated Probability	Probability Used in Simulation	Source
Meniscal repair	4%	4%	Ramme et al. [4]
Total knee arthroplasty	0–18%	2%	Mascarenhas et al. [38]; Yanke et al. [39]; McCormick et al. [40]
Reoperation	2–32%	12%	McCormick et al. [40]
“No issue”	71–85%	82%	Sgaglione et al. [41]
Successful repair	70–90%	80%	Sgaglione et al. [41]
Successful reoperation	-	100%	Ramme et al. [4]
Successful total knee arthroplasty	-	100%	Ramme et al. [4]
Repair to reoperation	-	20%	Ramme et al. [4]

**Table 3:** List of 3D-printed meniscus implant costs.

Item	Amount	Cost	Source
Stem cell		$1000	[43]
Cell culture	500 mL	$213	[44]
Human collagen-II	22.5 mL	$805.5	[45]
Alginate	5 g	$0.7	[45]
Polycaprolactone (PCL)	1 g	$0.4	[45]
TGF-β I	0.1 mg	$1076.5	[46]
ABS	63.5 g	$0.8	He et al. [47]
Acetone	10 mL	$0.1	He et al. [47]
Others (container, beaker, machine, electricity and labor cost etc.)	-	$27	He et al. [47]
Total	-	$3124	
